# Impact of Ventilator-Associated Pneumonia Preventative Measures and Ventilator Bundle Care in a Tertiary Care Hospital’s Adult Intensive Care Unit

**DOI:** 10.7759/cureus.59877

**Published:** 2024-05-08

**Authors:** Chandni Singh, Rashid Abdullah

**Affiliations:** 1 Department of Cardiac Anaesthesia, Laxmipat Singhania Institute of Cardiology, Kanpur, IND; 2 Department of Anaesthesiology and Critical Care, Chandni Hospital, Kanpur, IND

**Keywords:** ­trauma, tertiary care unit, bundle care, ventilator-associated pneumonia, cardiac intensive care unit

## Abstract

Background: The mitigation of ventilator-associated pneumonia (VAP) is a vital undertaking in safeguarding patient well-being. The research aimed to evaluate the impact of a multidisciplinary, comprehensive monitoring approach on VAP incidence in a tertiary medical-surgical-trauma critical care unit.

Methodology: The research was conducted within an adult medical-surgical ICU from June 2021 to December 2022. VAP data were collected by prospective targeted surveillance in accordance with the guidelines provided by the National Healthcare Safety Network (NHSN) and the Centers for Disease Control and Prevention. In contrast, a cross-sectional design was used to gather bundle data, according to the defined methodology of the Institute for Healthcare Improvement (IHI), and the rate of variation in admission prior to the bundle's installation was evaluated.

Result: The features of ventilated patients in adult medical-surgical ICUs were studied between 2021 and 2022. Regarding demographics, men comprised 42.6% and 45.3% of VAP patients and 65.3% and 50.7% of bundle care patients, respectively. Notably, 33.1% of patients in VAP and 54.5% in bundle care were over 60 years old. Clinical indicators such as median age (12.6 vs. 8 months for non-VAP vs. VAP patients), antibiotic usage (65% vs. 99% for non-VAP vs. VAP patients), and risk factors like trauma diagnosis (HR: 2.59, 95% CI: 2.07-3.23), and accidental extubation (HR: 4.11, 95% CI: 1.93-8.73) differed significantly between the bundle and non-bundle care groups. A significant increase in bundle compliance was seen from 90% in 2021 to 97% in 2022 (P-value <0.001), which helped to lower VAP rates and highlight the need for ongoing quality improvement in ICU treatment.

Conclusion: The use of ventilator bundles at a tertiary care hospital resulted in improvements in ventilator utilization, with an approximate increase of 20% and VAP rates of over 70% for adult critical patients.

## Introduction

Ventilator-associated pneumonia (VAP) is a common nosocomial infection that affects patients who are hospitalized in an intensive care unit (ICU) and are susceptible to pneumonia [1]. VAP is a significant complication arising from invasive mechanical ventilation (MV) and is the most often acquired infection in ICUs [2]. VAP is seen in a range of 9% to 24% of patients who have intubation for a duration beyond 48 hours [3]. This condition is linked to substantial morbidity and death rates, and it also results in a notable escalation in the utilization of resources [4,5]. The determination of whether a patient requires MV is contingent upon several factors, including the presence of comorbidities, the clinical condition of the patient, and the duration of MV [6,7]. Mathai et al. (2015) identified VAP as the prevailing nosocomial infection acquired by patients in the ICU [8].

Naboni (2021) examined the classification of VAP into two distinct types: early-onset VAP and late-onset VAP. Various risk factors have been identified that contribute to the increased mortality associated with VAP. These risk variables include advanced age, gender, prolonged MV, impaired consciousness, burns, the presence of comorbidities, previous antibiotic medication, invasive surgical procedures, and genetic polymorphisms [9]. Velásquez-Garcia et al. (2022) proposed that the incidence, etiological agents, and patterns of antimicrobial resistance associated with VAP vary across surgical ICUs in Western countries and developing Asian nations [10]. The prevalence of resistant organisms in Thailand has seen a gradual upward trend over the last decade [11].

However, there remains a significant gap in comprehensive understanding regarding the etiology of VAP and its implications, particularly within the ICUs of economically challenged Asian nations. This gap is particularly pronounced concerning infections stemming from drug-resistant organisms [12]. Parisi et al. (2016) conducted a study to evaluate the occurrence of VAP within a multidisciplinary critical care unit [12]. Their findings revealed an initial VAP rate of 21.6 per 1000 ventilator days. Subsequent to implementing interventions, a notable reduction in the occurrence rate was observed during the post-intervention period. Additionally, there was a significant decrease in the length of stay within the critical care unit, along with a reduction in the duration of MV [13].

Currently, the available evidence-based guidelines support the viability of avoiding VAP with the concurrent use of certain medications (proton pump inhibitors, sucralfate, and antimicrobial prophylaxis) [14,15]. This approach is sometimes referred to as a VAP bundle. Al-Dorzi et al. (2012) showed a decrease in rates of VAP through the implementation of active monitoring, the use of evidence-based preventative techniques, and comprehensive reporting [3].

Osman et al. (2020) observed occurrences of VAP in both the pre-bundle and bundle groups, with no significant difference between the two [16-18]. Patients undergoing MV are prone to various complications, including VAP, sepsis, acute respiratory distress syndrome, pulmonary embolism, barotrauma, and pulmonary edema. These complications often lead to prolonged periods of MV, extended hospitalizations in the ICU and hospital, increased healthcare costs, and heightened risk of morbidity and mortality. The mortality rates among individuals with acute lung injury undergoing MV vary depending on age, ranging from approximately 24% in younger patients to 71% in older patients [19-21]. The accumulation of contaminated secretions above the cuff of the endotracheal tube increases the susceptibility of patients to developing VAP [20]. Moreover, it correlated with a significant rise in the utilization of resources and healthcare expenditures. Majid et al. (2014) showed that implementing the IHI ventilator bundle in a tertiary care center resulted in a significant decrease of over 70% in VAP rates and a corresponding improvement of ventilator use by approximately 20% among adult critical patients [21]. Hence, preventing VAP in the ICU is widely acknowledged as a significant endeavor to ensure patient safety and enhance healthcare quality [22]. Multiple organizations have disseminated various solutions aimed at mitigating the occurrence of healthcare-associated pneumonia [23].

In this context, the present research endeavors to assess the impact of preventative measures and ventilator bundle implementation in an adult ICU setting of a tertiary care hospital in Kanpur, India, aiming to contribute to the ongoing efforts in VAP mitigation and patient care enhancement.

## Materials and methods

Two methodological approaches were used from June 2021 to December 2022 in the medical-surgical ICU of a tertiary care center in Kanpur. The collection of VAPs (pneumonia that develops in patients who are on MV for an extended period) data was carried out using a prospective surveillance approach according to the criteria and methods established by the National Healthcare Safety Network (NHSN) [4]. The data pertaining to bundles were gathered using a cross-sectional design in accordance with the methods established by the Institute for Healthcare Improvement (IHI) [2]. A skilled group followed national protocol to collect data on 300 ICU patients. The cohort consisted of 300 patients, divided into two groups: the patients with bundle care (n=150) and the non-bundle care group (n=150). The VAP preventative bundle followed in this study was a combination of evidence-based bundles [23]. The study monitored the occurrence of VAP among all patients receiving MV while also assessing the adherence to infection preventative bundles by collecting samples from ventilated patients and assessing the rate of VAP before implementation of the bundle.

The surveillance and assessment of VAP and compliance with infection control bundles were conducted intermittently in accordance with a risk-directed surveillance strategy. The focus was on monitoring and evaluating rather than implementing these measures.

Variables of the study

The study's independent variable pertains to real-time adherence to certain interventional approaches, such as the VAP bundle, among patients receiving MV.

Ethical concern

The study adhered to the ethical criteria set by the Institutional Ethical Committee of Chandni Hospital Pvt. Ltd. (ECR/1249/Inst/UP/2019).

Patients and setting

We used a non-probability purposive sample method to choose individuals who were receiving MV. The selection process included selecting patients who were admitted to the ICU within the designated time of data collection. This study focused on adult patients admitted to the University Hospital of Kanpur, a large tertiary hospital in Northern India. The specific criteria for inclusion in this study include ICU patients for a duration of 72 hours and required MV for 48 hours. A total of 800 individuals were subjected to examination inside the confines of a 10-bed ICU.

Data collection

A trained medical staff methodically collected data on a total of 800 patients admitted to the ICU, adhering to established national protocol requirements [3]. The cohort consisted of 300 VAP patients who were categorized into two groups: the individuals with bundle care (n=150) and the individuals without bundle care (n=150). The data included several elements such as demographic information, diagnoses, comorbidities, reasons for admission, SAPS II scores (simplified acute physiology score II), major procedures, interventions, and outcomes in the ICU and hospital settings. The recording of patients afflicted with infections encompasses several data points, including the date of onset, patterns of microorganism sensitivity, occurrences of repeated episodes, the underlying cause of infection, and the severity level. Each participant did routine validity checks to ensure the consistency, plausibility, and completeness of the data. Within this ICU, a team composed of professionals from many disciplines engaged in the process of validating data on a semester basis. This involved cross-referencing and verifying microbiological information by checking it against the comprehensive laboratory database.

Statistical analysis

The data were analyzed using IBM SPSS Statistics for Windows, Version 26 (Released 2019; IBM Corp., Armonk, New York) to assess the incidence of VAP, the following patient outcomes, and the prevalence of risk indicators at the time of ICU admission. The research looked at variations in the risk of VAP using multivariate logistic regression analysis. In order to account for potential confounding variables, such as age, type of hospitalization, and SAPS II score at the time of ICU admission, the research generated odds ratios and 95% confidence intervals. The study revealed a two-tailed P-value below the 0.05 threshold, indicating statistical significance.

## Results

This study involved a total of 300 patients from the ICU. The patients were categorized into two groups: the pre-bundle group (n=150) had VAP, and the non-bundle group (n=150) did not have VAP. The outcomes are as follows:

Demographic and clinical characteristics

Table 1 presents the demographic and clinical characteristics of ventilated patients admitted to the adult medical-surgical ICU between 2021 and 2022, focusing on two groups: "patients with bundle care" and "patients without bundle care" (referring to VAP patients). Regarding gender distribution, the majority of patients in both groups were male, constituting 511 and 15, respectively, and 410 and 6 females in each group, respectively. The distribution by age revealed a significant proportion of patients aged 60 and older in the bundle and non-bundle care groups.

**Table 1:** Demographic and clinical characteristics of VAP patients between 2021 and 2022 VAP: ventilator-associated pneumonia

Characteristics	Individuals With Bundle Care	Individuals Without Bundle Care
Gender (N)		
Male	511	15
Female	410	6
Age (years)		
Mean±SD	53.57±30.5	54.4±15.8
Groups N (%)		
18-39 years	227 (24.64)	3 (14.28)
40-59 years	259 (28.13)	6 (28.58)
60+ years	435 (47.23)	12 (57.14)

Comparison between VAP patients with bundle care and without bundle care

Table 2 presents a comparison between patients in the bundle care and without bundle care groups. The data are categorized based on various variables, including age, gender, underlying medical conditions, ventilation mode, and specific clinical indicators. The table displays the counts and percentages for each group, along with corresponding P-values, indicating the significance of the differences observed.

**Table 2:** Comparison between developed VAP patients bundle care and without bundle care groups VAP: ventilator-associated pneumonia; P-value: probability value; IQR: interquartile range

Variables	No VAP (n=62)	Developed VAP (n=33)	P-value
Median age in months	12.6	8	0.079
Male gender (%)	42 (67.74)	27 (81.81)	0.122
Underlying medical disease category (%)			
Metabolic/neurological (%)	9 (14.51)	6 (18.18)	-
Respiratory (%)	25 (40.32)	12 (36.36)	-
Oncology (%)	12 (19.35)	4 (12.12)	0.223
Immunodeficiency (%)	8 (12.90)	5 (15.15)	-
Cardiac (%)	8 (12.90)	6 (18.18)	-
Clinical factors (%)			
Endotracheal tube (%)	45 (72.5)	27 (81.81)	-
Tracheostomy tube (%)	7 (11.29)	1 (3.03)	0.307
High secretions (%)	19 (30.64)	6 (18.18)	0.002
High fraction of inspired oxygen (%)	23 (37.09)	22 (66.66)	0.000
High positive airway pressure (%)	13 (20.96)	32 (96.96)	0.000
High mean airway pressure (%)	12 (19.35)	10 (30.30)	0.000
High white blood cells (%)	21 (33.87)	32 (96.96)	0.022
High C-reactive protein (CRP)	22 (35.48)	21 (63.63)	0.000
Fever presence (%)	24 (38.70)	32 (96.96)	0.000
Antibiotic usage (%)	27 (47.54)	32 (96.96)	0.830
New infiltrates on chest X-ray (%)	23 (37.09)	28 (84.84)	0.000

The study found that 45% of patients required MV, with an average length of stay (LOS) of 3.5 days. Notably, patients who developed VAP had a median age of eight months (interquartile range (IQR): 30 months), while those who did not develop VAP had a median age of 12.6 months (IQR: 21 months). Furthermore, variables such as high secretions, high fraction of inspired oxygen, high positive airway pressure, high mean airway pressure, high white blood cell count, high C-reactive protein, fever presence, and new infiltrates on chest X-ray showed significant differences between the two groups. Notably, the VAP group exhibited higher proportions in these indicators compared to the non-VAP group. Additionally, antibiotic usage was notably higher in the VAP group (99%) compared to the non-VAP group (65%). These results underscore the clinical distinctions between patients who develop VAP and those who do not within the pre-bundle group, providing valuable insights for further analysis and considerations in patient care.

Risk factors in VAP

Table 3 presents the risk factors associated with the development of VAP using both multivariate Cox regression analyses. The multivariate analysis revealed several significant risk factors linked to an increased likelihood of VAP. Accidental extubation demonstrated a substantial risk, with a hazard ratio (HR) of 4.11 and a 95% confidence interval (CI) ranging from 1.93 to 8.73. Furthermore, trauma as a diagnosis compared to medical conditions showed an HR of 2.59 (95% CI: 2.07-3.23), indicating a significant risk factor. These findings underscore the importance of understanding and addressing these factors to mitigate the risk of VAP development in ICU patients. Accidental extubation, trauma diagnosis, COPD (chronic obstructive pulmonary disease), surgical admission, neuromuscular blockade use, and coma should be carefully considered and managed to improve patient outcomes and reduce VAP incidence.

**Table 3:** Pneumonia risk factors COPD: chronic obstructive pulmonary disease; CI: confidence interval; HR: hazard ratio; GCS: Glasgow coma scale; P-value: probability value

Characteristics	Univariate Cox Regression Analysis		Multivariate Cox Regression Analysis	
	HR (95% CI)	P-value	HR (95% CI)	P-value
Age (40-59 vs. 18-39 yr)	0.58 (0.51-0.73)	<0.001	-	-
Age (60 vs. 18-39 yr)	0.65 (0.56-0.83)	<0.001	-	-
Trauma vs. medical admission	2.45 (1.86-2.84)	<0.001	1.67 (1.08-2.13)	< .0001
Surgical vs. medical admission	2.34 (1.23-2.67)	0.19	2.55 (2.07-3.10)	0.04
COPD	1.09 (0.90-1.40)	0.68	1.41 (1.09-1.32)	0.05
Diabetes mellitus	0.42 (0.65-0.73)	<0.002	-	-
Hypertension (high blood pressure)	0.87 (0.70-0.70)	0.002	-	-
Neuromuscular blockade	2.37 (2.23-2.90)	0.001	1.45 (1.08-1.97)	0.03
Unplanned extubation	2.4 (1.43-5.11)	<0.001	3.12 (1.61-6.65)	<0.0001
Coma (GCS<9)	1.43 (1.14-1.72)	0.004	1.41 (1.12-1.72)	0.005

Comparisons of ventilator bundle compliance

**Figure 1:**
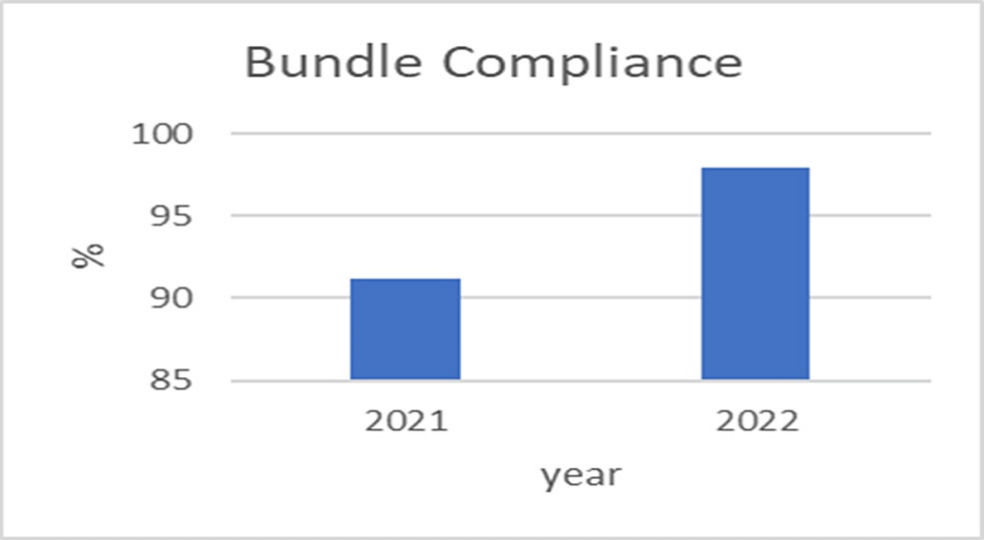
Ventilator bundle compliance between 2021 and 2022 Image credit: Chandni Singh

Figure 1 shows a significant increase in bundle compliance from 2021 to 2022. Bundle compliance measures adherence to a set of practices designed to prevent complications in patients on MV. The higher percentage in 2022 suggests that there was better adherence to these practices compared to the previous year.

**Figure 2:**
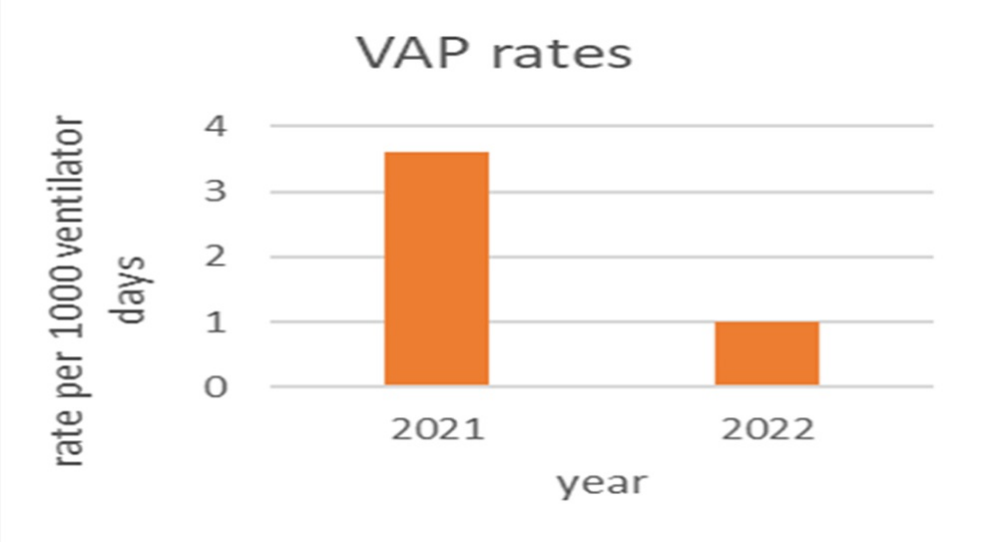
VAP rates between 2021 and 2022 VAP: ventilator-associated pneumonia Image credit: Chandni Singh

Figure 2 displays a decrease in the VAP rates from 2021 to 2022. VAP rates are a critical indicator of patient safety and quality of care in ICUs, reflecting the number of pneumonia cases per 1000 ventilator days. The reduction in VAP rates indicates improved patient outcomes and suggests that interventions or improved practices may have been effective.

**Figure 3:**
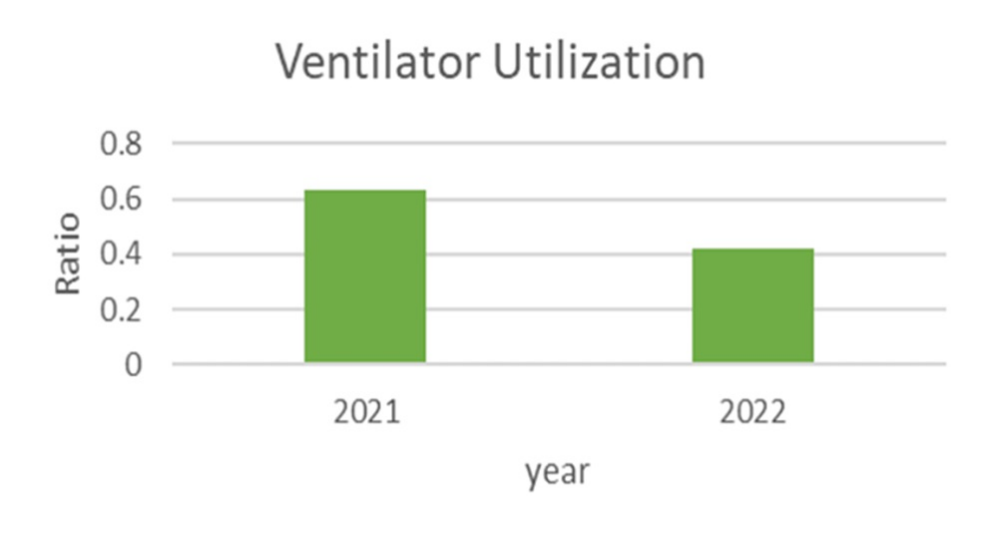
Ventilator utilization between 2021 and 2022 Image credit: Rashid Abdullah

Figure 3 compares the ratio of ventilator utilization between 2021 and 2022. The ratio has decreased slightly in 2022. This ratio is important for evaluating the usage of ventilators in a healthcare setting. A lower ratio can indicate either a reduced need for ventilatory support due to better overall patient health or even higher mortality. The data from these graphs suggest positive trends in the management of ventilated patients. Increased bundle compliance is likely contributing to the decrease in VAP rates. However, the cause of the decreased ventilator utilization is not immediately clear and requires further contextual information to be interpreted accurately.

These graphs collectively highlight the importance of continuous monitoring and quality improvement initiatives in healthcare settings. The findings showed a considerable improvement in ventilator bundle compliance from 90% in 2021 to 97% in 2022 (P-value<0.001).

Comparison of VAP rates between the studied ICUs and all NHSN ICUs

The ICUs in this study and all NHSN ICUs are compared in terms of VAP rates in Table 4. Medical, surgical, medical/surgical teaching, and other kinds of ICUs were among the ICU types included in the research sample. For each ICU type, the table shows the number of cases (n), the pooled mean VAP rate with standard deviation (SD), and the median VAP rate. Upon comparing the study sample with the NHSN data, it becomes evident that there are variations in VAP rates across different types of ICUs, providing valuable insights into the differences in VAP occurrence and severity between the ICUs in the study and the broader NHSN ICUs.

**Table 4:** Comparison of VAP rates between the ICUs in the study and all NHSN ICUs ICU: intensive care unit; SD: standard deviation; VAP: ventilator-associated pneumonia; NHSN: National Healthcare Safety Network

Factors	Study Sample			NHSN		
	n	Pooled mean (SD)	Median	n	Pooled mean (SD)	Median
Kinds of ICUs						
Medical (ICU)	64	1.3 (3.0)	1.22	82	2.0	1.8
Medical/surgical teaching	88	1.1 (2.3)	2.4	68	2.1	211
Surgical teaching	72	2.6 (3.2)	0	145	3.2	2.9
Surgical	51	3.4 (3.5)	4	79	4.1	5.6

## Discussion

Following a year of implementation, there was no discernible statistical shift in the VAP rate between the pre- and post-VAP preventative bundles in this research. According to the study, there was a gradual decline in VAP rates after the deployment of VAP bundles. This was likely caused by diligent monitoring and reporting. Several risk variables emerged as highly significant and should be the focus of future treatments. VAP is a significant nosocomial illness that is linked with considerable morbidity, death, and financial burden in healthcare settings. The prevalence of VAP varies between 10% and 25%, accompanied by a death rate ranging from 10% to 40%. The presence of VAP has been shown to result in an extended duration of hospitalization and an increase in hospital expenses [24].

Within the context of United States healthcare facilities, VAP has been identified as the second most financially burdensome nosocomial illness, with an estimated cost of $40,144 (95% CI: $36,286-44,220). Additionally, within underdeveloped nations, aggregate expenses associated with patients afflicted with VAP infections are roughly five times higher compared to patients without such illnesses [24]. This research assessed the effects of ventilator bundle care and preventative measures for VAP in an adult ICU in a tertiary care hospital. The data on VAP were collected using a prospective surveillance strategy according to the criteria and methodology outlined by NHSN [25]. The 300 patients in the cohort were divided into two groups: 150 patients in the bundle care group (n=150) who developed VAP and 150 patients without the bundle care group (n=150). The literature referred to the conformity to the VAP preventative package as a "game changer." Samra et al., for instance, looked at the efficacy of the bundle and adherence to it. They discovered that the incidence of VAP significantly decreased between the "without bundle care" and "bundle care" groups, with a minimum adherence rate of 94% per month (18.5% vs. 9%, P<0.05). The authors underlined that achieving "zero-VAP," which required close commitment to designating the bundle as a local policy and in-depth familiarity with the bundle's contents, took many months. Similarly, by recording both bundle compliance and VAP rate weekly, Caserta et al. found that after the compliance exceeded 95%, the rate of VAP reached nil within a few months. These findings suggest that although the compliance rate may skew the VAP rate, the bundle can effectively handle it, and its impact is not "Delphic." Therefore, we believe that achieving a higher compliance rate (up to 95%) would be crucial in attaining a VAP rate of 0%, even if only for a few months, as exemplified by the findings of Samra et al. and Caserta et al. [26,27].

VAP's univariate and multivariate Cox regression risk variables are shown in Table 3. The use of neuromuscular blockade during ICU stays, unconsciousness, COPD, and inadvertent extubation were all associated with an increased risk of VAP, according to multivariate analysis. According to Bornstain et al. [28] and de Lassence et al. [29], there is a correlation between this and the following: odds ratio (OR), 3.2; 95% CI: 1.3-7.9; and relative risk, 5.3 (95% CI: 2.8-9.9; P<.001). As a patient safety measure, extubation accidents must be prevented; however, since these incidents are uncommon, the total VAP rate is probably going to be relatively unaffected by these kinds of interventions. The VAP rate increased by 38% to 85% with the deployment of an IHI ventilator bundle, according to a recent analysis of eight trials (from 2004 to 2009) that used the bundle either alone or in conjunction with other strategies to avoid VAP in ICUs [29]. Furthermore, a 56% decrease in the VAP rate was linked to the installation of many preventative measures, including the IHI bundle's components, in 44 ICUs across 13 developing nations. Table 3 compares VAP rates between the study's ICUs and all NHSN ICUs. The research comprises medical, medical/surgical teaching, medical/surgical other, and surgical ICUs. The table displays the number of cases (n), pooled mean VAP rate with SD, and median VAP rate for each ICU category. Comparing the research sample to NHSN data shows that VAP rates vary among various kinds of ICUs, revealing disparities in VAP prevalence and severity across the study's ICUs and the NHSN.

The present research used the IHI VAP bundle, which led to a significant drop in the VAP rate and sustained this decrease for a duration of 12 months. Based on the findings of Tawfiq et al. (2010), the use of a VAP preventative bundle led to a decrease in VAP rates. Specifically, the mean number of VAP cases per 1000 ventilator days decreased from 9.3 in 2006 to 2.3 in 2007 and further to 2.2 in 2008 (P<.001) [25]. Additionally, an analysis of yearly metrics revealed a significant increase in ventilator bundle compliance, with rates rising from 90% in 2021 to 97% in 2022 (P<0.001). The study found that VAP occurred in 42% of the pre-bundle group and 25% of the bundle group. Using a P-value of 0.650%, the statistical analysis suggested no significant difference in VAP incidence between the bundle care and without bundle care groups. Furthermore, the incidence rates of VAP were calculated to be 22 per 1000 ventilator days in the pre-bundle group and 16 per 1000 ventilator days in the bundle group. This information indicates the prevalence and rates of VAP in the study population, showcasing the potential effect of the intervention bundle on VAP occurrence.

In the study conducted by Majid et al. (2014), a zero rate of VAP was attained toward the conclusion of the research. It is important to note that the long-term durability of these zero rates requires more monitoring and observation. The attainment and maintenance of zero rates or rates near zero are feasible but require ongoing surveillance and steadfast adherence to a preventative package with compliance rates above 95% [21]. Despite the vigorous implementation of preventative bundles, many comprehensive intervention programs were unable to attain a VAP rate of zero. This phenomenon may be partly attributed to host variables that are not subject to modification. In addition, it is worth noting that regulations requiring the public disclosure of VAP rates may have inadvertently led to a more cautious interpretation of subjective indicators used to define VAP. This cautious approach may have artificially lowered the reported rates of VAP. However, research has demonstrated a significant increase in compliance with ventilator bundle protocols, rising from 90% in 2021 to 97% in 2022 (with a chi-square test yielding a P-value of less than 0.001).

The present research had many notable limitations, including the extensive examination of ventilator days, prolonged length of investigation, and meticulous ascertainment of VAP occurrences by an infectious disease consultant. Secondly, due to the nature of our study design, we are unable to establish a causal link between the variables examined. Additionally, the use of ecologic analysis, which involves analyzing aggregate data rather than individual patient data, may introduce ecological bias and limit our ability to draw conclusions about patient-based outcomes.

## Conclusions

The results of this research cast doubt on the usefulness of using the VAP preventative bundle to lower the VAP rate and urge further prospective, multicenter studies to evaluate the bundle's efficacy. There was an estimated 20% reduction in ventilator use among adult critical patients. The results indicate that a high level of compliance with the bundle method is necessary to effectively avoid VAP. Upon implementing a ventilator bundle, adult critical patients at a tertiary care center showed improvements in ventilator use of around 20% and VAP rates of over 70%. It is evident that the mere implementation of a strategy is inadequate for effectively mitigating VAP rates. The implementation of treatments and monitoring of bundle compliance are essential components for achieving a reduction in rates. Further investigation is necessary to further our understanding of this area. Implementing a comprehensive and well-supported program has the potential to significantly improve the efficacy of therapies, particularly when combined with strict adherence to the VAP bundle and a reduction in the incidence of VAP among MV patients.
